# The search for new materials and the role of novel processing routes

**DOI:** 10.1007/s43939-021-00014-y

**Published:** 2021-06-07

**Authors:** Peter J. Wellmann

**Affiliations:** 1 European Materials Research Society (E-MRS), 23 Rue du Loess, Strasbourg, France; 2 International Union of Materials Research Societies (IUMRS), Singapore, Singapore; 3 Crystal Growth Lab, Department of Materials Science & Technology 6, Friedrich-Alexander University Erlangen-Nürnberg (FAU), Martensstr. 6, Erlangen, Germany

## Abstract

Throughout human history, most further developments or new achievements were accompanied by new materials or new processes that enabled the technologic progress. With concrete devices and applications in mind, synthesis and subsequent treatment of materials naturally went along with the progress. The aim of the underlying article is to spot the role of optimization, of discovery, of trial-and-error approaches, of fundamentals and curiosity driven design and development. In a consecutive examination, five missions addressing the challenges facing our world (identified by the European Council) will be cross linked with seven topical areas from materials science defined by the European Materials Research Society. The scope of this examination is to identify approaches and methods to further develop and innovate materials which form the basis of the anticipated solutions.

## Application and curiosity driven search for new materials

Human history and the origin of today’s culturally and technologically advanced civilization are closely related to the continual progress in terms of the usage of new materials and the application of innovative processing routes (see e.g. [1, 2] for further reading on the history of materials developments). Phrases like “materials science”, “materials engineering” or “materials technology”, however, are rather new. The development of materials was closely linked to applications and concrete devices that were needed in daily life. For example, to realize new tools, new materials from the environment were chosen that usually needed new handling routines to fabricate the envisaged device like a knife. In a progressing step, base materials were mixed and by subsequent treatment using drying and heat new properties were found. The driving force for materials developments was strongly pushed by the application in mind (see e.g. [3–5] for further reading on the history of technology since the ancient times and their relation to the evolution of today’s civilization). Archeological findings of various tools, devices of the daily life and hunting weapons, respectively, bear witness of the first processing technologies of metals, ceramic materials or even glass, to name a selection of a few long-term stable examples. Besides very practical application areas that made daily life easier, one may attribute several achievements to pleasure, to wellbeing or to art, to name a few. To spotlight a quite specific item, cave painting driven by the inner stimulus to visualize and to preserve events from daily life was enabled by the development of dyes. A quite important role in materials development played the human power of observation. For example, magnetic materials have been discovered, they were not designed. Magnetite, iron oxide that was magnetically polarized in the earth's magnetic field, was found to attract iron. Another strong driving force to develop new materials was and still is related to military hardware. Throughout human history numerous examples document that a predominance in weapon technology went and still goes hand in hand with a novel and advanced materials processing technology. Nevertheless, a lot of efforts in materials development were and are related to peaceful use like for medical care and for environmental protection.

While perhaps most of the materials developments were triggered by concrete applications in mind, also curiosity driven search and attentive observation used to play and still plays an important role (see [6] for examples of discoveries and curiosity driven developments in history). Hence, an active strategy to develop new materials that serves the human civilization in a wholistic manner may be sectioned into (a) application driven and (b) curiosity driven motivations.

## Engineering and natural science driven approach in the search of new materials

**Fig. 1:**
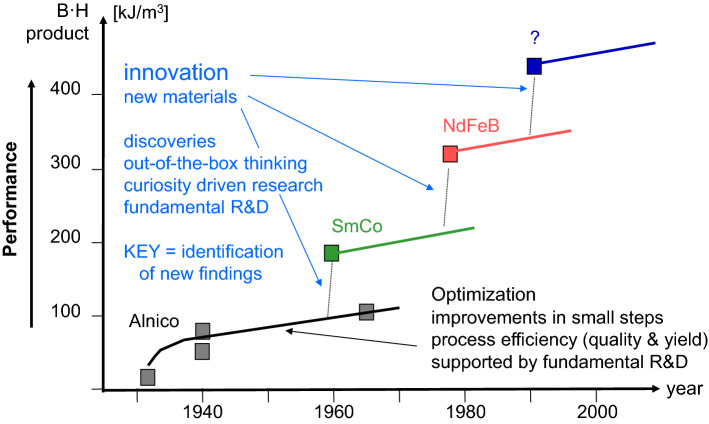
Illustration of the evolution of materials performance related to optimization and innovation. Exemplary, the evolution of hard magnetic materials is shown as they are indispensable in performance electrical transportation, wind energy and mechatronics in general. The so-called B·H product represents the magnetic energy stored in the materials and directly reflects the magnetic field flux that can be realized in a magnetic device. Significant performance improvements stem from new materials through innovation. Still, optimization is necessary to further improve the applicability of the new materials as well as the processing yield. Magnetic property data were taken from [7]

Coming from the application driven approach to develop new materials, engineering is a probate attempt to improve materials properties like hardness, fracture behavior, temperature stability and in general environmental resistance, to grasp a few aspects [1]. Such developments are often based on a kind of iterative trial-and-error procedure as well as modifications of the processing in small steps. The advantage of this approach is obvious: Although not knowing all underlying fundamentals, by observation and stringent conclusions new processes and new materials exhibiting superior properties have been developed. Such a procedure may be called the optimization approach. A significant portion of the technology development is based on such a strategy. Numerous examples show that optimization improves the properties of materials and devices, but at a certain high level a stagnation will be reached. A further improvement will be impossible by optimization. At this point, thinking outside-of-the-box that includes an innovation step is the only way to reach a higher level. To give an example: In the case of hard magnetic materials [7], Alnico (alloy out of Aluminum, Iron, Nickel and Cobalt) exhibiting a cubic crystal lattice is a performance alloy from the early and mid-twentieth century. It was successively improved over several decades by optimization of the material composition and the processing technology that influenced the microstructure. Finally, the so called BH-product, which is a key figure of merit of hard magnetic materials, stagnated at around 45 kJ/m^3^. Physically, this limitation is linked to the highly symmetric crystal lattice with a medium magnetic crystal anisotropy constant. By optimization of the processing, for instance, the pronounced needle-like morphology added a magnetic shape anisotropy component and hereby improved the hard magnetic properties. Nevertheless, this approach was limited. The innovative step was initiated by switching to the newly designed material Samarium Cobalt (SmCo_5_) exhibiting a uniaxial, hexagonal crystal lattice that intrinsically exhibits a greater magnetic crystal anisotropy constant than Alnico. The typical BH-product of Samarium Cobalt of ca. 200 kJ/m^3^ is four to five times greater than in the case of Alnico, respectively (see Fig. 1). This example illustrates that a deeper fundamental understanding of the materials properties fosters innovation. Therefore, fundamental research in natural sciences like physics, chemistry and biology forms a backbone for innovation. In this context it is noteworthy that numerous innovations stem from discoveries. This basically includes unexpected results and observations. Several times, discoveries went ahead with experiments that were firstly interpreted as failures. Owing to the openminded monitoring of the unexpected data, a discovery was made, or an innovation was triggered.

In anticipation of Sect. 5, high throughput experimental methods, in which materials composition and processing parameters are screened for the search of new materials, may be interpreted as a systematic and artificial approach to trigger discoveries. Computer simulation in materials science on all scales, starting at an atomistic level, considering the meso and micro scale up to the macroscopic device and application, offers an additional tool to identify novel materials exhibiting superior properties. Data driven approaches that progressively include algorithms from machine learning and artificial intelligence significantly bias innovation in materials science (further reading e.g. [8–11]).

## The discipline of materials science

Although materials developments have been performed since the beginning of human civilization, compared to the natural sciences, philosophy and theology, respectively, materials science is a new discipline that has been established mainly since the midst of the twentieth century.

Up to date, numerous materials developments are initiated by the application in mind. Metallurgical processing and fabrication of ceramics are prominent examples how materials further-developments were motivated from manufacturing processes.

Since the late nineteenth and within the twentieth century, the newly established chemical industry has brought up a large variety of synthetic materials which exhibit novel properties that have become indispensable for many applications.

With the establishment of solid-state chemistry and physics since the beginning of the twentieth century, increasingly fundamental materials research has been carried out in applied research divisions of universities, research institutes and industrial development centers.

Triggered by this progress, during the past thirty to fifty years more and more materials research departments have been founded around the globe that work interdisciplinary between (1) manufacturing engineering, (2) natural sciences (physics, chemistry, biology) and (3) medicine.

Meanwhile, several study courses in the “discipline” of materials science have been established that have the same popularity as the well-known natural sciences, manufacturing and electrical engineering, respectively. The concrete study courses may vary significantly from university to university. In fact, the International Standard Classification of Education (ISCED-F-2013) published by the UNESCO Institute for Statistics [12] lacks a detailed field description of a study course on materials science. There is a global need for the standardization of a kind of basic curriculum related to a study course in materials science and technology including functional materials.

## Materials research societies and scientific exchange beyond borders and political systems

Throughout the twentieth and the early twenty-first century, academic science has been connecting researchers around the world beyond ideologic and political systems. Since the mid 1970ies, a number of materials research societies around the globe are serving the scientific and engineering community by bringing together experts from physics, chemistry, biology, medicine/pharmacy and engineering in general. As a rough estimation, in the range of 40,000 to 50,000 participants from industry, government, academia and research laboratories meet annually to share recent progress in functional materials science. Although the majority of the conference attendees usually stems from the same region as the materials research society, basically all meetings attract attendees from all over the world. Due to the special pandemic situation related to COVID-19, almost all larger meetings in the years 2020 and 2021 are carried out as virtual meetings. This new kind of model may be transformed in future time into truly hybrid meetings where conference attendees meet onsite and through remote. Such a new model may even foster the global attendance of the various meetings.

In the global context, in the year 1991 the International Union of Materials Research Societies (IUMRS) was founded that connected numerous regional materials research societies from the Americas (MRS (USA), Mexican-MRS), from Europe (European-MRS), from Asia (Chinese-MRS, MRS-Taiwan, MRS-India) and Australia (Australian-MRS). Today, IUMRS is continuously seeking to gather all global regions to coordinate the outreach in materials science and to assist to develop a lively materials community. Although IUMRS had to face the one or the other setback in recent years, the society is continuously pursuing its mission to serve the scientific community worldwide.

## A prospective global view on functional materials science

Materials science includes all kind of materials classes in terms of properties and processing technology. Nevertheless, the abovementioned materials research societies by trend are looking into functional materials science.

To exemplify the topical areas of significant global interest, a compilation assembled by the E-MRS will be presented. In the years 2019 and 2020 seven topics have been selected which will be fostered within the next five to ten years. These topics are linked with the five missions that have been pointed out by the European Council [13–15] for the upcoming years of the current decade: (i) Adaptation to Climate Change including Societal Transformation, (ii) Cancer, (iii) Healthy Oceans, Seas, Coastal and Inland Waters, (iv) Climate-Neutral and Smart Cities and (v) Soil Health and Food (see Table 1).

**Table 1:** Crosslinking of the 7 topical materials research areas of significant global interest and the five missions that have been pointed out by the European Council for the upcoming years of the current decade

Number of asterisks indicate impact of the tropical areas on solutions within the missions	Mission 1 Adaptation to climate change incl. societal transformation	Mission 2 Cancer	Mission 3 Healthy oceans, seas, coastal and inland waters	Mission 4 Climate-neutral and smart cities	Mission 5 Soil health and food
Tropical areas					
(1) Bio and nanomedical materials	*	***	*	**	**
(2) Materials for sensing and embedded systems	***	***	***	***	***
(3) Advanced battery materials and processing/energy storage materials (including hydrogen and green algae)	**	*	***	***	*
(4) Materials for quantum information	**	*	*	***	*
(5) Materials and processes for additive manufacturing	**	**	*	***	*
(6) Resources, recycling and sustainable material development	***	**	***	***	***
(7) Artificial intelligence (AI) in materials science research	***	***	***	***	***

The number of asterisks emphasize on a basal engineering understanding the by trend expected applicability of the development of new materials related to the topical areas 1 to 7 on the expected solutions within the missions 1 to 5: *expected punctual applicability, ** expected common applicability, *** expected broad applicability. Independently of this estimation, even a punctual applicability may reach a very high relevance for the society

These missions have been identified looking at the needs the global society is facing. Certain areas may have additional specific targets, nevertheless, the missions are of common high value. In almost every case, materials form the basis to realize devices in distinct applications. The seven topical areas identified by the E-MRS aim to serve the materials research society in a prospective way as focal points, albeit the tasks within each area are much broader than the presented exemplification is able to outline.

1. Bio and nanomedical materials: In recent years nanoparticles have been identified as drug delivery systems. Nanoparticles may carry the medical component in their interior, while functional molecules may be added to the surface for a selective delivery inside the human body. Hereby, drugs can be precisely delivered to certain organs. Since nanoparticles and many biological molecules have approximately the same dimensions, a manifold of interactions is possible. Other medical applications of nanomaterials target contrast agents, analytic tools and diagnostic devices. The related therapies are of particular interest for cancer therapy. In addition, chronic infective diseases profit from potentially selective and long-term agent delivery properties. Dialysis support or even replacement by nanomedicine is intensively discussed. Materials scientists are sought to share their skills in nanotechnology in interdisciplinary bio-medical teams.
2. Materials for sensing and embedded systems: Semiconductors, thin film coatings and functionalized surfaces are the backbone of most sensor devices. In the broader context of the Internet of Things (IoT), sensors comprise the physical sensing device that measure a physical property or the concentration of chemical or biological molecules. The embedded system supplies the computer hardware for processing of the measured data in the context of the desired analysis as well as for communication with the higher-level control unit. The sensing and the controller unit make use of high-quality semiconductor devices. Besides the standard Si material, wide bandgap semiconductors play an increasingly important role. Among others, SiC proves to be a chemical inert and a radiation hard material that may be applied in harsh environments as gas sensor, optical detector or X-ray detector. Another new material for sensing applications is graphene. While there are several processing routes for graphene, it is worth to mention that graphene can be grown epitaxially on SiC by sublimation of silicon from the semiconductor surface. The latter fact could enable integrated sensors based on graphene as sensing unit and SiC as electronic interface towards the data acquisition system.
  Sensors (sensing device plus embedded system) illustrate how the key topical areas exhibit applications in several of the five missions described above. Semiconductor sensors that detect gases, air composition, air pressure and temperature, respectively, are important in all five missions: They may be applied to detect climate changes (mission i), they enable the diagnoses of diseases (mission ii), they reveal the state of the environment (mission iii), they enable the control of air conditioning systems (mission iv) and they may identify the ecologic status of farmland (mission v).
3. Advanced Battery Materials and Processing/Energy Storage Materials (including hydrogen and green algae): Recently, the phrase “green technology” is increasingly used to point out its sustainability and its compatibility from an ecologic perspective. The electric grid turns out to play a key role, especially if a positive CO_2_ balance in energy provision and consumption is envisaged. In this context, electric battery technology and power electronics for electric transportation are key enablers. The further development of battery technology needs interdisciplinary R&D activities that include novel materials, interfaces in an electrochemical context as well as advanced processing technologies. Again, the abovementioned graphene as well as related carbon allotropes could play an important role as electrode and component of the anode. In the case of power electronics, the wide bandgap semiconductors silicon carbide and gallium nitride are in the process of replacing the standard semiconductors.
  While short-term and to a certain amount also mid-term storage of electrical energy may be performed using batteries, mid- and long-term storage needs other solutions. So called “green” hydrogen produced by electricity from photovoltaics and wind energy is widely discussed as a key player for energy storage. Technical solutions in this context need tremendous efforts in materials studies. Here, the various materials research societies around the globe, together with the IUMRS offer the platform to bring together experts from all regions to share and discuss their new findings.
4. Materials for quantum information: Materials for quantum information aim to provide new solutions in secure information transmission and more powerful computation. Instead of the binary data system and the Boolean algebra, quantum states (so called qbits) and novel quantum state related algorithms will be used. From the technical point of view, solutions based on solid state electronics are favorable. Materials that enable long spin coherence times of the excited states are needed. Materials research in this area focuses on superconducting materials, quantum structures of semiconductors that exhibit high charge carrier mobilities and long charge carrier diffusion lengths, interfaces at magnetic layers including thin metal films and hybrid magnetic semiconductors. In addition, optically or electrically accessible excited states of point defects in semiconductors that again exhibit long spin coherence times are widely studied. Although some of the concepts under discussion are already two or even three decades under investigation, the research field is still in its initial stage and continuous efforts for the search of new materials and better processing routes are necessary. Such efforts need joint actions of researchers from materials science and physics.
5. Materials and processes for additive manufacturing: Additive manufacturing allows a completely new degree of freedom in the structural design of devices. To exemplary foreground 3D printing, beside the necessity to provide new precursors out of particles, liquids and filaments, respectively, there is also a need for advanced processing routes. One benefit of additive manufacturing is related to the flexible way of prototyping and even series production of complex or sophisticated structures. Metal-, polymer- and ceramic-based devices, respectively, have already been realized. The saving of resources in the manufacturing process is striking. Beyond this, additive manufacturing enables new functionalities that are impossible with the traditional construction and assembly procedures. Large hollow cores, reduced weight and improved stiffness or bending properties may be realized. Such efforts need a close collaboration between the disciplines of materials science and manufacturing.
6. Resources, recycling and sustainable material development: The limited availability of resources of base materials and energy as well as the necessity of waste prevention are the major driving forces to recycle materials from devices that reached their lifetime. Recycling itself must be supported by a smart device design that enables the separation of the base materials in an economic and ecological manner. In an ideal case, the manufacturing process exhibits a low consumption of base materials as well as energy to run the process. In this context, there are two major tasks materials scientist and manufacturing engineers need to focus on. Optimization of each step of the process is inevitable. In addition, some processing routes must be thought in a completely new manner. Innovative approaches like the above-described additive manufacturing need to be applied and groundbreaking methods need to be invented. Basically, each processing step needs to consider in new ways using an out-of-the-box development approach.
7. Artificial intelligence (AI) in materials science research: High throughput experimental and computer simulation create a tremendous data set that needs systematic data storage and data analysis efforts. Computer algorithms based on machine learning and artificial intelligence concepts are needed to handle that valuable amount of data and to draw the right conclusions. As desktop computers revolutionized the lab and office work in research, concepts of machine learning and artificial intelligence will become a new toolset for carrying out experiments and for examining complicated and tremendous data sets. It is inevitable that materials research societies offer as a cross-disciplinary action an educational platform including tutorials and networks to train researchers of all ages.

To work on this perspective future in materials science, the role of the regional material research societies is to bring together experts from interdisciplinary fields to identify solutions for the various problems. First, the platform for a scientific and technology-driven dialogue between experts from specific fields needs to be provided. Second, interdisciplinary exchange needs to be initiated. Experts from science and technology need to be gathered who exchange their novel ideas and who are willing to learn from each other. The role of IUMRS is to provide a platform that enables a strong interlink of the regional materials research societies in the global network.

## Summary

The provision of performance materials and related new processes form the basis for all technologic solutions to encounter the global challenges adjusting to climate changes, fighting cancer and pandemic, protecting of the seas, living in greener cities as well as safeguarding of soil health and food. One may summarize the necessary actions in materials science and engineering to be taken using 3 lemmas.

The realization of higher performance devices and applications depends on.

i. Innovation followed by optimization of novel materials and processes.
ii. Curiosity driven research and development that encounters fundamentals from materials science and that interdisciplinary merges natural and engineering sciences.
iii. Classic and still very valuable tools that are accompanied by high throughput methods which foster discoveries as well as by the implementation of algorithms from artificial intelligence and machine learning which act as completely new analysis tools.
